# Aneurysmal Rupture of a Mesodiverticular Band to a Meckel's Diverticulum

**DOI:** 10.1155/2015/603064

**Published:** 2015-01-21

**Authors:** Christian Sommerhalder, Kenneth R. Fretwell, Gregory G. Salzler, John M. Creasy, R. Jonathan Robitsek, Sebastian D. Schubl

**Affiliations:** ^1^Ross University School of Medicine, St. Kitts, Dominica; ^2^Department of Surgery, Jamaica Hospital Medical Center, Jamaica, NY 11418, USA; ^3^Department of Surgery, Weill Cornell Medical College, New York, NY 10065, USA

## Abstract

Aneurysmal rupture of a mesodiverticular band has not previously been reported in the clinical literature. We are reporting a case of hemoperitoneum in a 51-year-old male after an aneurysmal rupture of a mesodiverticular band. This case demonstrates that in rare instances, a rupture of the mesodiverticular band leading to Meckel's diverticulum can lead to significant hemoperitoneum. This is usually caused by a traumatic injury but in our case was apparently caused by an aneurysm of the mesodiverticular artery. Patients with known Meckel's diverticula should be aware of the possibility of rupture, as should clinicians treating those with a history of this usually benign congenital abnormality. Rapid surgical intervention is necessary to repair the source of bleeding, as massive blood loss was encountered in this case.

## 1. Introduction

Meckel's diverticulum is the most common congenital malformation of the GI tract, occurring in between 0.6% and 4.0% of the population [1]. Most patients with Meckel's diverticulum remain asymptomatic, with a very low probability (<2%) of becoming symptomatic or experiencing complications (4.2%–6.4%) over their lifetime [2]. Cases of Meckel's diverticulum are most commonly discovered incidentally, and removal of a symptomatic Meckel's diverticulum is accepted practice. However, it remains a matter of debate whether asymptomatic Meckel's diverticula should be resected [3–6]. Bleeding complications from Meckel's diverticulum are almost exclusively due to acid production by ectopic gastric mucosal tissue found in the diverticulum, causing small bowel ulcer formation and intraluminal blood loss. Very rare cases of diverticular perforation are reported, but these generally do not present as hemoperitoneum. In approximately 10% of patients with Meckel's diverticulum, the blood supply originates not from the mesentery of the distal small bowel, but from a separate vessel originating from the superior mesenteric artery in what is known as a mesodiverticular band [7, 8]. In our patient, no perforation of the diverticulum or the opposing intestinal wall was found. Instead, an apparently spontaneous rupture of the mesodiverticular artery caused significant hemoperitoneum that was later discovered to have resulted from an underlying aneurysmal dilation of the vessel.

## 2. Case Report

A 51-year-old male presented to Jamaica Hospital Medical Center for severe abdominal pain and nausea that began abruptly during a five-hour transcontinental flight. His past medical history included only asthma. Upon arrival at the hospital, he developed recurrent nonbloody, nonbilious emesis. His initial vital signs included a blood pressure of 133/75 mmHg, pulse of 83 BPM, and temperature of 36.3°C. A physical exam revealed rebound tenderness and guarding. His initial labs were as follows: white blood cell count of 15.3 K/*μ*L, hemoglobin of 15.7 g/dL, hematocrit of 47.8%, platelet count of 376 K/*μ*L, and a normal coagulation profile. The patient stated that the day prior to the flight, he had a solid bowel movement with no blood or melena noted and was not vomiting at that time; he also denied any sick contacts.

**Figure 1:**
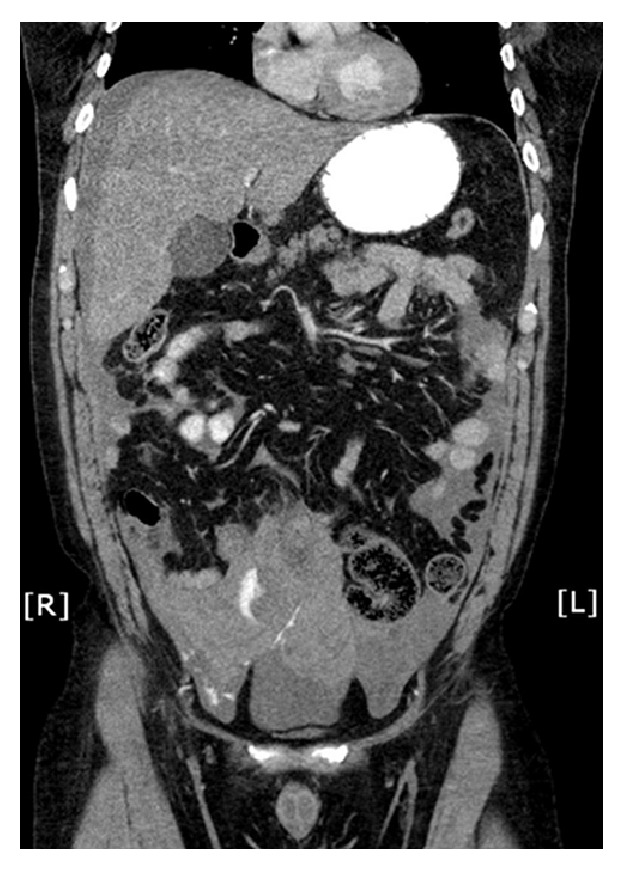
Coronal view of contrasted CT demonstrating the hemoperitoneum with contrast blush.

After several hours in the Emergency Department, the patient attempted to stand and lost consciousness. An immediate blood pressure was noted to be 53 systolic. Repeat blood work was sent which revealed a decreasing hematocrit (48% to 30%) and hemoglobin (15.7 g/dL to 9.6 g/dL). Fluids were started and an emergent abdominal CT was performed. The CT scan revealed hyper-dense fluid present throughout the abdomen and pelvis, with heterogeneity of the fluid and an active contrast blush (Figure 1). A surgical consult was called and the patient was taken to the operating room emergently for exploration.

In the operating room, Meckel's diverticulum was encountered with a mesodiverticular band that appeared to have torn, resulting in massive intraperitoneal hemorrhage. A small bowel resection was performed to include Meckel's diverticulum, and continuity was reestablished with a side-side stapled enteric anastomosis. Total blood loss for the case was 2000 mL; 3 units of packed red blood cells and 3 units of fresh frozen plasma were administered. The patient was transferred to the SICU postoperatively and subsequently discharged on postoperative day 8.

## 3. Histology

**Figure 2:**
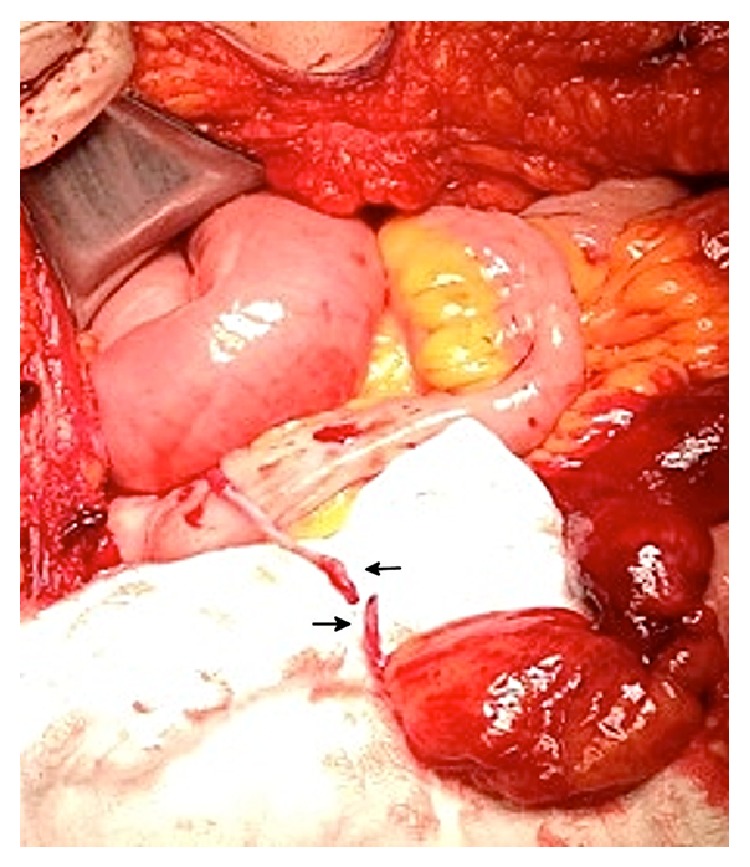
Rupture of the persistent vitelline artery. The top arrow points to the mesenteric side of the vitelline artery, while the lower arrow shows the artery attached to the fat globule of Meckel's diverticulum.

**Figure 3:**
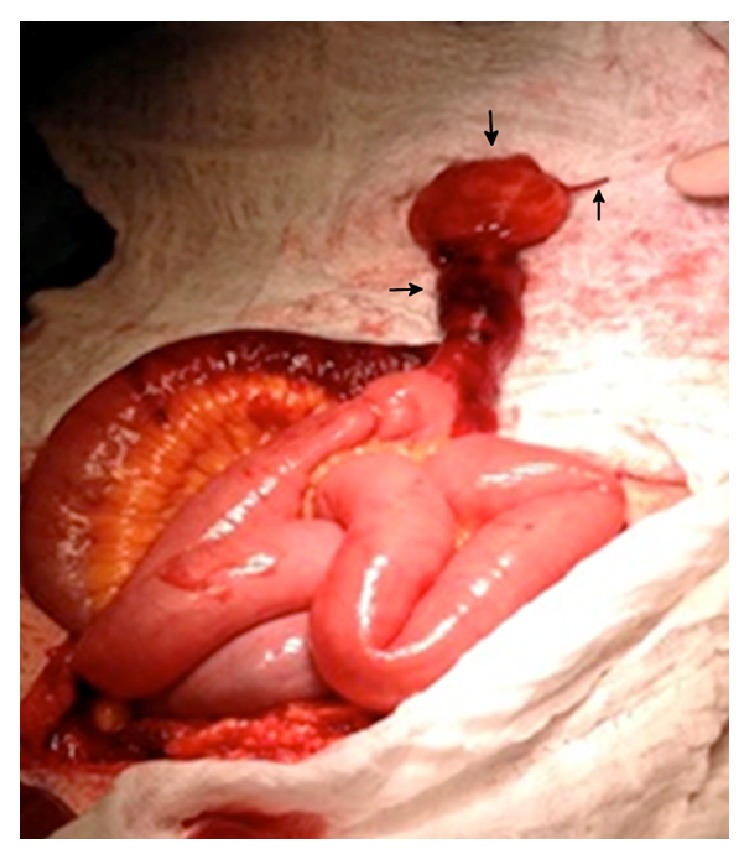
Meckel's diverticulum (left arrow) with fatty tissue distally (top arrow) and ruptured vitelline artery (right arrow).

On gross examination, the diverticulum appeared to be completely intact and measured 7.5 cm in length and 4.0 cm in diameter. At the tip of the outpouching there was surrounding fatty tissue (Figures 2 and 3), and through this tip of fat there was a tubular structure resembling a vessel measuring 2.5 cm in length and 0.1 cm in diameter. On microscopic examination, the small bowel segment showed an acute ischemic injury to the mucosa with transmural hemorrhage and myocytolysis. The mesodiverticular band itself showed an intimal hyperplasia and elastotic degeneration of the adventitia with evidence of an aneurysmal rupture.

## 4. Discussion

During normal embryonic development, the left and right vitelline arteries originate from the primitive aorta; the left normally involutes and the right becomes the superior mesenteric artery. In cases where Meckel's diverticulum remains, a branch of this vitelline artery can result in a direct blood supply to the diverticulum through the mesodiverticular band [9]. Cases of small bowel obstruction or strangulation due to this mesodiverticular band are relatively common [10–12], but ruptures leading to hemoperitoneum are extremely rare. Based on the pathology report, it appears as though the mesodiverticular vessel became aneurysmal, leading to rupture. The exact timing of the rupture in our case is unknown, but it is possible that the initial abdominal pain was due to an obstruction or strangulation of the bowel by the mesodiverticular band, with subsequent rupture of the aneurysm. Three of four prior case reports describe traumatic injury as being responsible for rupture of a mesodiverticular band. In two cases, rupture was due to a car accident [13, 14], and in one case rupture resulted from abdominal trauma during a softball game [15]. In the final instance, division of the band occurred during reconstruction of an aneurysmal abdominal aorta and right common iliac artery [16]. In our patient, it appears as though the aneurysm burst either spontaneously or as a result of some tension on the mesodiverticular band due to a bowel obstruction, most likely prior to the syncopal episode.

The histology of the actual diverticulum in our case showed simple columnar intestinal mucosa. Meckel's diverticulum may contain various histologies including normal ileal tissue (26%) or have abnormal histology including ectopic tissue (43%), diverticulitis (25%), and enteroliths (6%) [6, 17]. Although ectopic gastric mucosa was initially thought to be the most common tissue type, more recent studies suggest that gastric mucosa is found in 20–33% of symptomatic patients, and as few as 8.3% of nonsymptomatic patients [6, 17]. Other histologic variants include pancreatic tissue, carcinoid, duodenal, lipoma, and leiomyosarcoma. Meckel's diverticulum can lead to various complications such as GI bleeding, obstruction, intestinal perforation, and cancers. The most common complication among both children and adults is intestinal obstruction, though some studies suggest that bleeding is most common in children [6, 17]. Although the incidence of Meckel's diverticulum is more or less equal in males and females, males tend to have a higher incidence of these complications, with an overall decline in risk with increasing age [1, 2, 18].

## 5. Conclusion

This is the first reported case of a mesodiverticular band rupturing due to an aneurysm of the arterial vessel. Three PubMed indexed case reports have been written on a rupture of the mesodiverticular band due to trauma and one instance where it was injured during an operation. As of the writing of this report, no PubMed indexed cases were found that report a spontaneous rupture of the mesodiverticular band. These rare events can rapidly lead to significant hemoperitoneum and in delayed or severe cases potentially result in patient demise. They should be managed with immediate surgical intervention if a diverticulum with hemoperitoneum is suspected on imaging or by patient history.
